# The Risk of SARS-CoV-2 in Immunosuppressed IBD Patients

**DOI:** 10.1093/crocol/otaa026

**Published:** 2020-04-16

**Authors:** Peter D R Higgins, Siew Ng, Silvio Danese, Krishna Rao

**Affiliations:** 1 Department of Internal Medicine, University of Michigan, Ann Arbor, Michigan, USA; 2 Department of Internal Medicine, Chinese University of Hong Kong, Hong Kong, China; 3 Department of Internal Medicine, Humanitas University, Milan, Italy

## Abstract

This is a rapid review of the risk of infection with SARS-CoV-2, the risk of symptomatic cases of COVID-19, and the severity of these cases in IBD patients. Guidance on how to manage IBD patients at risk for infection, and IBD patients after infection is provided. The prevention of infection in both patients and health care providers by reducing elective visits and procedures, utilizing telemedicine, and social isolation is also emphasized to maintain health care services for IBD patients during a growing pandemic.

## Lay Summary

The SARS-CoV-19 virus and COVID-19 disease raise important questions about the risks to immunosuppressed IBD patients. We review the available data, including the SECURE IBD registry, and draw parallels to previous pandemics of influenza, SARs, and MERS.

## Graphical Abstract

**Graphical Abstract:**
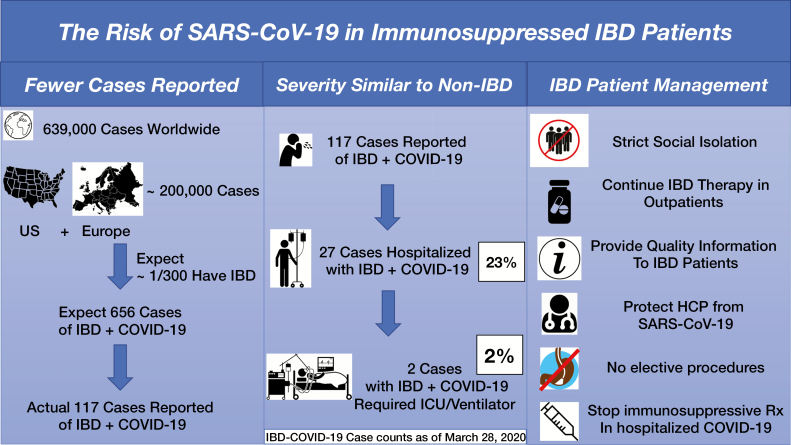

Since the first case of COVID-19 disease was diagnosed in Wuhan, China, in December, 2019, the SARS-CoV-2 virus has spread across the globe, infecting more than 600,000 people by April 1, 2020, the date of this review. Both physicians and IBD patients have been concerned that the immunosuppressant medications that we use to great effect in IBD patients could put them at risk for both more frequent infections by SARS-CoV19, and more severe manifestations of the COVID-19 disease.

## GLOBAL EXPERIENCE WITH COVID-19 IN IBD

Contrary to this expectation, initial reports from both China and Northern Italy suggested a lower infection rate in IBD patients compared with the general population. IBD patients were under-represented in those diagnosed with symptomatic COVID-19 disease in both locations. It is possible that IBD patients with COVID-19 are being under-tested and under-reported compared with people without IBD. However, the fact that patients on immunosuppressants are generally more likely to seek health care and get tested for symptoms, and more likely to be regarded with concern by health care professionals, compared with people without IBD makes this possibility unlikely.

An international registry, SECURE-IBD,^1^ was formed under the auspices of the IOIBD, which has been tracking reported cases since March 15, 2020. At the time of this report, 239 cases, with only 7 receiving artificial ventilation, and 11 deaths have been reported in IBD patients in association with COVID-19 disease. Based on a population prevalence of 1 IBD patient for every 300 persons in the population, we would expect about 683 cases in the United States and 1487 cases in Europe at the time of this report (total 205,000 cases in the United States, and 446,000 in Europe), yielding a theoretical deficit of 1931 cases of COVID-19 disease in IBD patients in the United States and Europe.

The Pediatric Porto Group has reported 8 cases of COVID-19 in children with IBD,^2^ all of which were mild. In China and South Korea, no cases of COVID-19 in pediatric IBD have been reported, but biologic treatment was delayed in 79 children, with 17/79 (22%) having flares of IBD during a pandemic.

## THE SEVERITY OF SARS-COV-2 IN IBD

It was reasonable to be concerned that SARS-CoV19 infection in IBD patients could be more severe, particularly in those patients on immunosuppressive medications. While we have limited numbers of cases reported in the SECURE-IBD registry at the time of this report, most cases were mild, and only 7 were admitted to ICUs. One hundred fifty of the 239 reported cases were on biologic or combination therapy, including 113 on anti-TNF therapy, 21 on anti-integrin therapy, and 16 on anti-IL-12/23 therapy. An additional 16 were on maintenance JAK inhibitor therapy for their IBD. Of the 7 admitted to ICUs, all received ventilator therapy. Of the 16 deaths, 1 was never hospitalized, and 31 more did not reach ICU-level care, which could indicate rapid clinical progression among those who died without ICU care, or that their local health care system was overwhelmed with a surge of COVID-19 patients. At the time of this report, 176/239 (74%) of cases of COVID-19 in IBD were being treated as outpatients, and few had their IBD therapy discontinued after infection.

## INSIGHTS FROM MILAN AND HONG KONG

Italy currently has more than 105,000 cases of COVID-19, with over 12,000 deaths, but only 13 reported COVID-19 cases in IBD patients, all of which were mild (personal report, Silvio Danese). The spread of SARS-CoV-2 throughout Italy has been explosive, producing overwhelming numbers of patients requiring inpatient and ICU care. More than 60 physicians in Italy have died following coronavirus infection.

Hong Kong has 765 confirmed cases of COVID-19, with only a few patients on immunosuppressants. The first case of COVID-19 was diagnosed in Hong Kong on 23 January and since then the government has rapidly responded (having learned painful lessons during the SARS epidemic 17 years ago), including social isolation (schools closed since February). Early identification, adequate testing, contact tracing, and quarantine have largely succeeded in controlling the spread of SARS-CoV-2.

## LESSONS FROM SARS, MERS, AND INFLUENZA FOR IBD IN A TIME OF PANDEMIC

Both SARS-CoV-1 and Middle Eastern Respiratory Syndrome (MERS-CoV) were much more likely to produce symptoms in everyone infected, making case identification much simpler than with SARS-CoV-2 infection, which has many asymptomatic and mild cases. Quarantining confirmed cases proved very effective in stopping the spread of both SARS-CoV-1 and MERS-CoV infection. Extensive testing of all patients with symptoms and all contacts of proven cases to identify all cases of SARS-CoV-2 infection, followed by strict quarantine will be a critical piece of the ongoing public health response to the COVID-19 pandemic.

Steroid therapy was extensively tested in the treatment of severe SARS-1, and proved of no benefit, and possibly produced harm. SARS-1 notably produced severe inflammation, with high levels of IL-6, TNF-ɑ, interferons, and multi-organ system failure, and case reports suggest patients with this level of inflammation are benefitting from anti-IL6 monoclonal antibody therapy, prompting randomized clinical trials testing tocilizumab and sarilumab.

MERS spread rapidly among travelers in 2012, particularly those making the Hajj to Mecca, and had a case mortality rate over 34%. Despite high temperatures in the Middle East, MERS-CoV is highly transmissible, with over 19% of cases in health care workers, and still occurs in ~10 cases per month, largely in Saudi Arabia, with seasonal increases in the warmest months of the year.

A case–control study of the 2009 pandemic of H1N1 influenza found that infection was not more frequent in IBD patients on immunosuppressive medications, though the incidence of H1N1 was 7 times higher in young IBD patients. Vaccination for H1N1 in IBD patients in 2009 produced seroprotection in 85% of non-IBD patients, but only 64% in IBD patients not being immunosuppressed, and only 36% in those on combination therapy.^3^

The 1918 H1N1 pandemic began in the spring of 1918, but had its highest number of fatalities in a second wave in the fall of 1918, and a 3rd wave in the spring of 1919 caused roughly 25% of the total deaths, suggesting that multiple waves of this pandemic are possible.

## MANAGEMENT OF IBD PATIENTS DURING THE PANDEMIC

### Social Isolation and Soap

It is important to reinforce the public health messaging of strict social isolation in the time of a pandemic, and this is especially important for patients who are immunosuppressed. IBD patients should minimize contact with others, stay at home, avoid group gatherings of any size, use frequent handwashing, and utilize any food delivery or medication delivery services available to them to minimize contact with others. Effective handwashing and decontamination of surfaces is important for IBD patients, and with evidence that SARS-CoV-2 can be found in stool, this may be particularly important in shared bathrooms to prevent fecal-oral transmission. Minimizing clinic and hospital visits through the use of telemedicine is advisable. Elective endoscopic procedures should be postponed.

### Continue IBD Therapy in Outpatients with IBD

It is important for patients to avoid IBD flares, especially severe flares that could require steroids. It is believed that steroids will increase the risk of coronavirus infection, and having a flare that requires hospitalization will likely put patients at a higher risk of SARS-CoV-2 infection. The risk of IBD flares due to discontinuation of medications, even if this occurs in only 5–20% of patients, is important, as there will be few opportunities for hospitalization in a context of overloaded hospitals filled with COVID-infected patients. Keeping off of steroids such as prednisone/prednisolone is advised, if possible, as studies show no benefit in viral illnesses and possible harm. The risks and benefits of continuing or stopping IBD therapy should be carefully weighed,^4^ as the current pandemic could last several months.

### Provide Information, and Combat Misinformation and Disinformation

It is helpful to provide accurate information to all IBD patients, as inaccurate information on the internet can lead to inappropriate use of speculative therapies, including prophylactic use of hydroxychloroquine, which has led to a shortage for patients with rheumatologic disease and at least one overdose with chloroquine. Physicians can play an important role in providing accurate information to IBD patients.

### Protect Health Care Providers

We need to stop elective procedures and focus on controlling this pandemic. If we lose too many providers to illness and quarantine, we will not be able to handle a surge of severe COVID-19 cases. For in-person clinics and urgent endoscopy cases, we should check temperature, symptoms, and travel history in all patients and accompanying persons. If either person is at risk, we should divert them to a fever clinic to evaluate for COVID-19 and refill their IBD medications to prevent a flare.

Adequate Personal Protective Equipment (PPE) is essential to maintaining an effective health care workforce. Endoscopy providers should have enhanced training in donning and doffing full PPE and procedural attire for urgent endoscopic cases, which should include hair covering, face shield/goggles, respirator, shoe covers, gown, and gloves. Cases with a high risk of transmission should be deferred if possible, and if not possible to defer, these should be done in a negative pressure room with staff wearing full PPE. Endoscopy units should minimize patient cross-infection with thorough environmental cleaning between cases.

Health care providers should routinely wear masks in health care settings, do extended hand washing, and practice social distancing, including avoiding social events (including department meetings) with colleagues. Clinic visits should be converted to virtual visits by telemedicine or telephone where available. Rotating health care workers in cohorts, divided by specialty, can be helpful in avoiding a loss of expertise due to illness and quarantine after exposure. For example, if all of the advanced endoscopists at a center become infected with SARS-CoV-2, services for pancreaticobiliary disease will be severely limited for at least 14 days. Thoughtfully rotating providers can sustain services.

### Stop Immunosuppressive IBD Therapy for 7–21 Days for Symptomatic COVID-19 Patients

Most IBD therapies have a long biologic half-life, so that a short pause during hospitalization for infection is unlikely to lead to a flare. There is some risk of developing antibodies after a pause in therapy, but the clinical trajectory of a case of COVID-19 disease is often clear within 7 days of presentation, and patients who are recovering well can have their therapy restarted. Detailed recommendations on individual medications from the International Organization of IBD (IOIBD) have been posted online.^5^

## CONCLUSIONS

Early data suggest that patients with IBD, including those on immunosuppressive therapies, are not having symptomatic COVID-19 infection at higher rates than the non-IBD population, and it is possible that there are fewer COVID-19 cases in IBD patients. This could be due to either undertesting or underreporting of IBD cases vs non-IBD cases, or to IBD cases being milder than non-IBD cases due to anti-inflammatory therapy, or to genetic differences in pulmonary inflammation.^6^ The severity of COVID-19 infections in IBD patients, when they occur, appears largely similar to non-IBD patients, with most cases in the mild category, with only 23% of cases requiring hospitalization, and only 2% requiring ICU care. Continued tracking of IBD patients with symptomatic COVID-19 through the international SECURE-COVID data repository will continue to enlighten us about the effects of SARS-CoV-2 on IBD patients.

Our experience with MERS suggests that summer temperatures may not temper this coronavirus pandemic, and the 1918 H1N1 influenza outbreak suggests that multiple waves of infection could occur within the first 18 months. Vaccinations against SARS-CoV-2, when available, could be less helpful in IBD patients on immunosuppression (to be determined), based on our experience with the 2009 H1N1 influenza vaccine. It is important to remember that all pandemics end, and this one will be no exception. Many of the recommendations above will be critical in protecting our IBD patients and providers and learning how to prepare for the next one, as we weather this storm and wait for its eventual passage.

## Notes

Supported by National Institutes of Health (NIH) grant R01 DK118154.
